# Global trends of research on depression in breast cancer: A bibliometric study based on VOSviewer

**DOI:** 10.3389/fpsyg.2022.969679

**Published:** 2022-09-26

**Authors:** Ling Chen, Tingting Ren, Yun Tan, Hong Li

**Affiliations:** Department of Hepatobiliary Surgery, Affiliated Hospital of Guizhou Medical University, Guiyang, China

**Keywords:** breast cancer, bibliometric analysis, co-citation analysis, research hotspots, depression

## Abstract

**Background:**

Depression is common psychiatric morbidity in breast cancer survivors, seriously affecting patients’ quality of life and mental health. A growing body of research has investigated depression in breast cancer. However, no visual bibliometric analysis was conducted in this field. This study aimed to visualize the literature to identify hotspots and frontiers in research on breast cancer and depression.

**Methods:**

The publications related to depression in breast cancer were retrieved in the Web of Science Core Collection between 1 January 2002 and 17 March 2022. VOSviewer was used to identify co-occurrences and collaborations among countries, institutions, and keywords. CiteSpace was used to detect keyword bursts.

**Results:**

A total of 7,350 articles and reviews related to depression in breast cancer were identified. From 2002 to 2022, the United States and the People’s Republic of China were the most productive countries in this field. The University of California, Los Angeles, and the University of Toronto were the most productive institutions in this field. The Journal of Psycho-oncology, followed by Supportive Care in Cancer and the Journal of Clinical Oncology, had the most publications on depression in breast cancer. Of the top 10 journals, seven were from the United States, two were from England, and one was from Germany. Five research hotspots of depression in breast cancer were identified by co-word analysis. Research on post-traumatic growth, spiritual interventions, cognitive-behavioral therapy, physical activity, and symptom cluster management of depression in breast cancer was relatively mature in the core hotspots. Burst detection of keywords on depression in breast cancer showed the latest hotspots, such as mental health, cancer survivor mortality, and activity.

**Conclusion:**

The research on depression in breast cancer is growing. Attention should be paid to the latest hotspots, such as mental health, cancer survivor, mortality, exercise, and physical activity.

## Introduction

Breast cancer is the most prevalent malignant tumor in women, and its incidence is increasing yearly. Cancer statistics from the American Cancer Society (ACS) have shown that breast cancer is the most common cancer in women and the second leading cause of death, with increasing incidences yearly (Casadonte et al., 2021). According to recent data from the International Agency for Research on Cancer (IARC), the World Health Organization Agency for Cancer, there were approximately 2.26 million new female breast cancer cases worldwide in 2020, accounting for a quarter of all new cancer cases among women (Ferlay et al., 2021). Depression is a mental disorder that affects mental well-being, occupational performance, and quality of life, leading to physical symptoms. Depression is prevalent in 32.2% of all countries (Kulczyński et al., 2019). Of all malignant tumor sufferers, patients with breast cancer have the most severe psychological problems, with 40% of patients experiencing mental health problems (Oo et al., 2021). Surgery is an intense stressor for breast cancer patients and may destroy female secondary sexual characteristics. Therefore, the psychological stress of breast cancer patients during the perioperative period is particularly prominent, manifested as increased heart rate, elevated blood pressure, nervousness, anxiety, fear, sleep disturbance, etc. It can result in decreased immunity and affect the operation and postoperative rehabilitation. Radiotherapy and fatigue bring an excessive economic burden to the psychological burden. Lack of understanding from society and family, discrimination, and patients’ psychological endurance all contribute to depressive symptoms in patients.

Bibliometric analysis is an emerging tool for quickly exploring the status and trends of a subject or domain *via* statistical methods and visualization. This approach identifies relevant nodes and extracts useful information from large volumes of information (Jiang et al., 2018; Wang et al., 2021). Bibliometric software has been used in the scientometric analysis. CiteSpace and VOSviewer are the most popular tools for visualizing and analyzing scientific literature trends and patterns in bibliometric analysis (Wang and Lu, 2020; Xie et al., 2020).

So far, few bibliometric studies have been conducted on the global development of depression in breast cancer. To better understand the global trends and hot topics of depression in breast cancer, we conducted a bibliometric analysis of global publications related to depression in breast cancer between 2002 and 2022 based on VOSviewer.

## Materials and methods

### Data source

The data of this study were extracted from the Web of Science Core Collection on 17 March 2022, using the following retrieval strategy: “Breast Neoplasm*” OR “Breast Tumor*” OR “Breast Cancer” OR “Mammary Cancer*” OR “Malignant Neoplasm of Breast” OR “Human Mammary Neoplasm*” OR “Malignant Tumor of Breast” OR “Breast Malignant Tumor*” OR “Human Mammary Carcinoma*” OR “Breast Carcinoma*” (Topic) and Depress* (Topic), with the timespan from 2002 to 2022. Only articles and reviews were included. The language was limited to English. Two researchers independently retrieved and screened the publications. Any disagreement was resolved through discussion.

### Data extraction

The following results of the literature were evaluated and recorded: (1) publication year, (2) journal title, (3) total citation count, (4) authorship, (5) WoS category, and (6) manuscript type. These manuscripts’ full records and cited references were imported into VOSviewer (CWTS, Leiden University, Leiden, Netherlands) for bibliometric analysis.

VOSviewer (Leiden University, Leiden, Netherlands) is a free visualized software for creating accessible maps using bibliographic data. VOS stands for “Visualization Of Similarities.” CiteSpace, designed by Professor Chaomei Chen, is a freely available Java application that can visualize and analyze scientific literature trends and patterns. This study used CiteSpace V and VOSviewer 1.6.8 to identify top journals, institutions, countries, co-occurrence of keywords, co-cited articles, and trends.

## Results

### Analysis of annual publications

**Figure 1:**
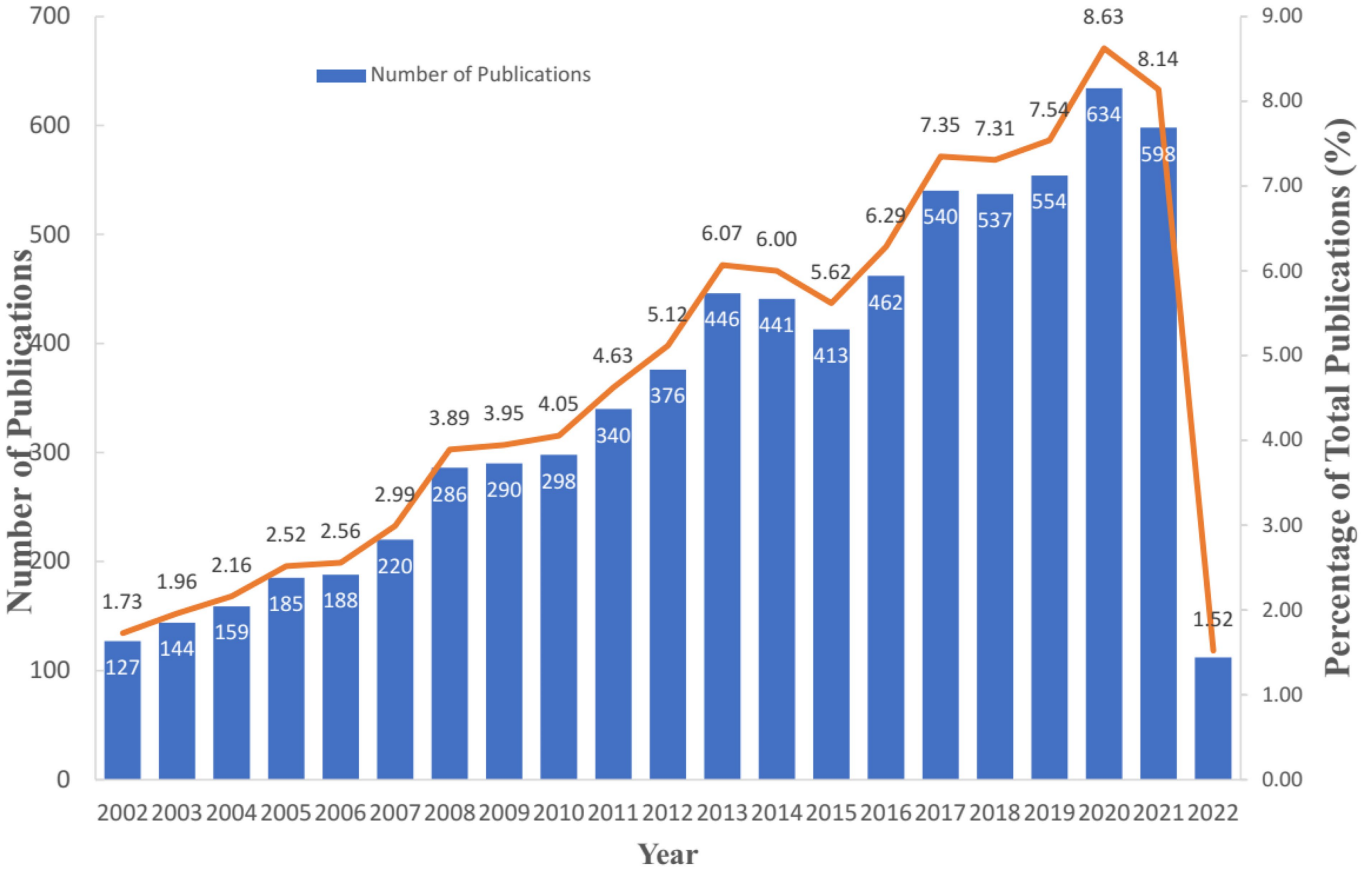
The annual publications on depression in breast cancer from 2002 to 2022.

From 2002 to 2022, 7,350 articles and reviews about depression in breast cancer were published in the Web of Science database (Figure 1). In the first 10 years, the topic of depression in breast cancer showed steady growth, accounting for 36.66% of the total publications. In the last 10 years, the number of papers on depression in breast cancer grew rapidly. As of 17 March 2022, 112 articles about depression caused by advanced breast cancer have been published, which are expected to overgrow throughout 2022. This indicates that depression in advanced breast cancer is gaining increasing attention.

### Distribution characteristics of author

**Figure 2:**
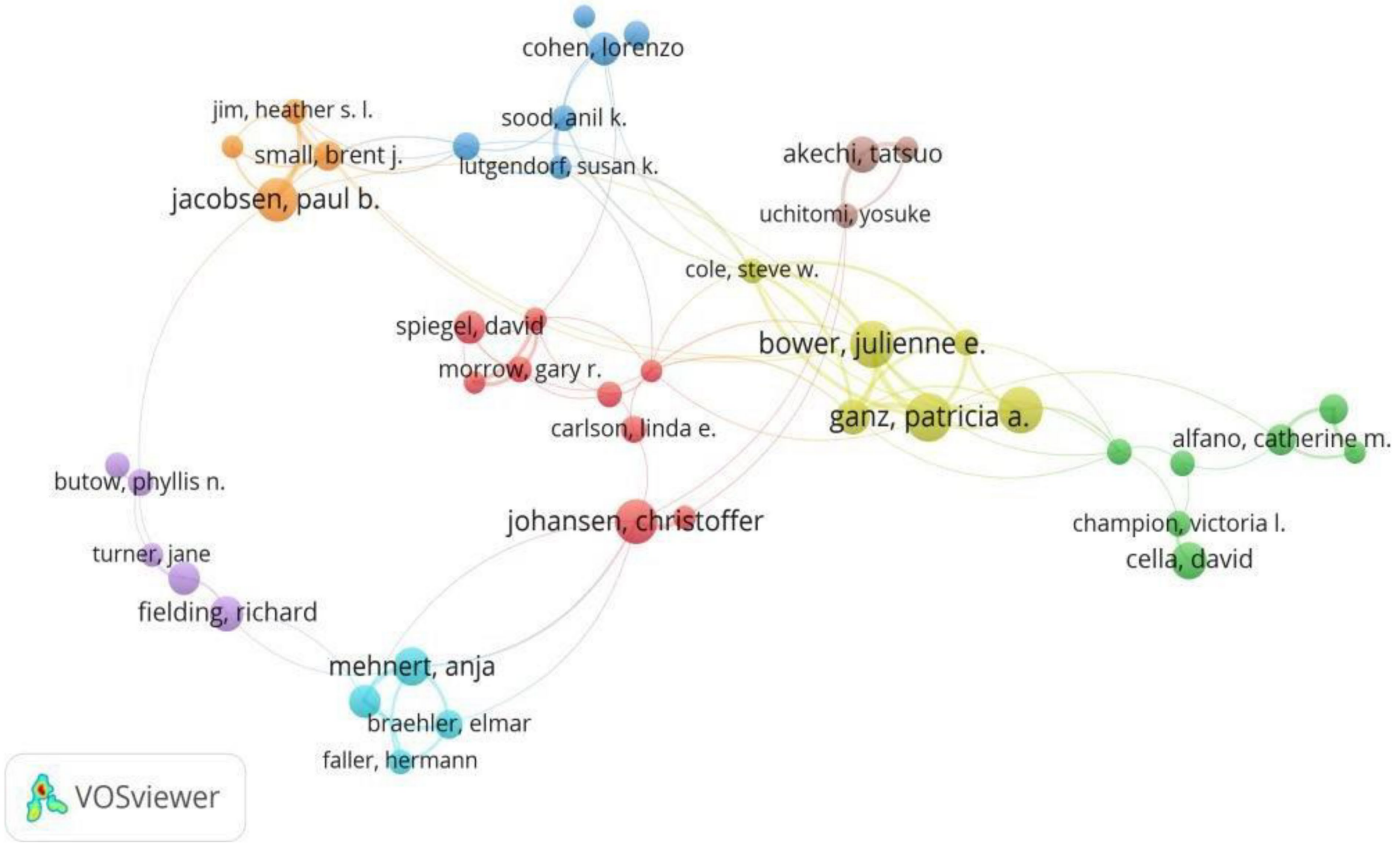
A visualization network of collaboration between authors on depression in breast cancer.

A total of 32,630 authors with 7,350 publications were involved in this field. The top 10 productive authors and co-cited authors are listed in Table 1. Miaskowski from the University of California in the United States was the most prolific author (69 publications), followed by Paul Steven (49 publications) and M Ganz Particia (43 publications). The authors’ co-cited network was generated as shown in Figure 2. Zigmond As was the most frequently co-cited author (1,445 citations), followed by Bower Julienne (1,380 citations) and Ganz Particia (990 citations).

**Table 1:** Top 10 productive authors and co-cited authors in the field of depression in breast cancer.

Rank	Author	Counts	Rank	Co-cited author	Citation
1	Miaskowski	69	1	Zigmond As	1,445
2	Paul Steven M	49	2	Bower Julienne	1,380
3	Ganz Patricia	43	3	Ganz Patricia	990
4	Bower Julienne	41	4	Carson Le	763
5	Stanto Annette L	41	5	Spiegel D	710
6	Johansen Christoffer	38	6	Radloff Ls	672
7	Jacobsen Paul	37	7	Beck At	666
8	Cooper Bruce	33	8	Stanton	620
9	Mehnert Anja	31	9	Watson	614
10	Evine Jon	30	10	Mitchell	594

### Analysis of countries/regions and institutions

**Figure 3:**
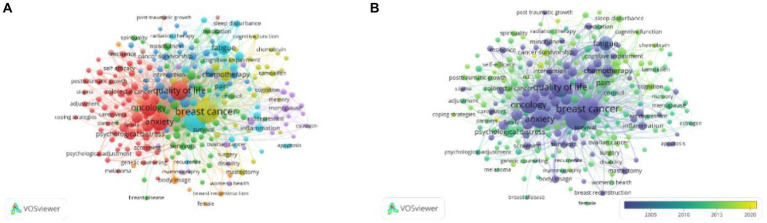
A visualization network of collaboration between countries and regions.

Sixty-four countries/regions have been involved in the studies of depression in breast cancer since 2002. The top 10 countries published 6,195 articles over the last two decades (Table 2). The United States was the most productive country (2,632 publications) with the highest H-index (221), followed by China (644 publications) with an H-index of 75, and England (479 publications) with an H-index of 126. As shown in Figure 3, the intensive clusters between China and the United States in the collaborative network indicated close cooperation between the two countries. The University of California made the most outstanding contributions to this field (169 publications), followed by the University of Toronto (144 publications), University of California (143 publications), and Memorial Sloan Kettering Cancer Center (135 publications).

**Table 2:** The main countries, regions, and institutions contributing to publications on depression in breast cancer.

Rank	Country/region	Counts	Proportion(%)	H-index	Institutions	Counts	Proportion(%)	Citations
1	United States	2,632	35.81%	221	Univ Calif Losangeles	169	2.3	11,422
2	The Peoples Republic Of China	644	8.76%	75	Univ Toronto	144	1.96	6,990
3	England	479	6.52%	126	Univ Calif San Francisco	143	1.95	5,566
4	Australia	472	6.42%	104	Mem Sloan Kettering Canc Ctr	135	1.84	8,682
5	Canada	471	6.41%	138	Univ Texas Md Anderson Canc Ctr	131	1.78	5,669
6	Germany	439	5.97%	114	Univ Pittsburgh	119	1.62	5,002
7	Netherlands	357	4.86%	102	Univ Sydney	106	1.44	3,887
8	South Korea	267	3.63%	58	Univ Michigan	102	1.39	4,582
9	Italy	235	3.20%	66	Dana Farber Canc Inst	99	1.35	4,619
10	Japan	199	2.71%	64	Duke Univ	97	1.32	4,279

### Analysis of journals

The top 10 productive journals with over 2,000 publications are listed in Table 3. The Journal of Psycho-Oncology ranked first with 730 publications (9.93%), followed by Supportive Care in Cancer (444, 6.04%) and Journal of Clinical Oncology (164, 2.23%). Of the top 10 journals, seven were from the US, two from England, and one from Germany. The Journal of Clinical Oncology had the highest impact factor (44.544), followed by Cancer (6.86).

**Table 3:** The top 10 journals related to depression in breast cancer.

Ranking	Name	Country/region	Number of publication	% of total publication	Impact factor (2020)	H-index (2020)
1	Psycho Oncology	United States	730	9.93	3.894	132
2	Supportive Care in Cancer	Germany	444	6.04	3.603	84
3	Journal of Clinical Oncology	United States	164	2.23	44.544	145
4	Cancer	United States	150	2.04	6.86	90
5	Breast Cancer Research and Treatment	United States	146	1.99	4.679	48
6	Cancer Nursing	United States	146	1.99	2.592	65
7	Journal of Pain and Symptom Management	United States	126	1.71	3.612	66
8	BMC Cancer	England	109	1.48	4.43	42
9	European Journal of Cancer Care	England	103	1.40	2.52	38
10	Oncology Nursing Forum	United States	102	1.39	2.172	56

### Co-occurrence analysis and clustering analysis of keywords

**Figure 4:**
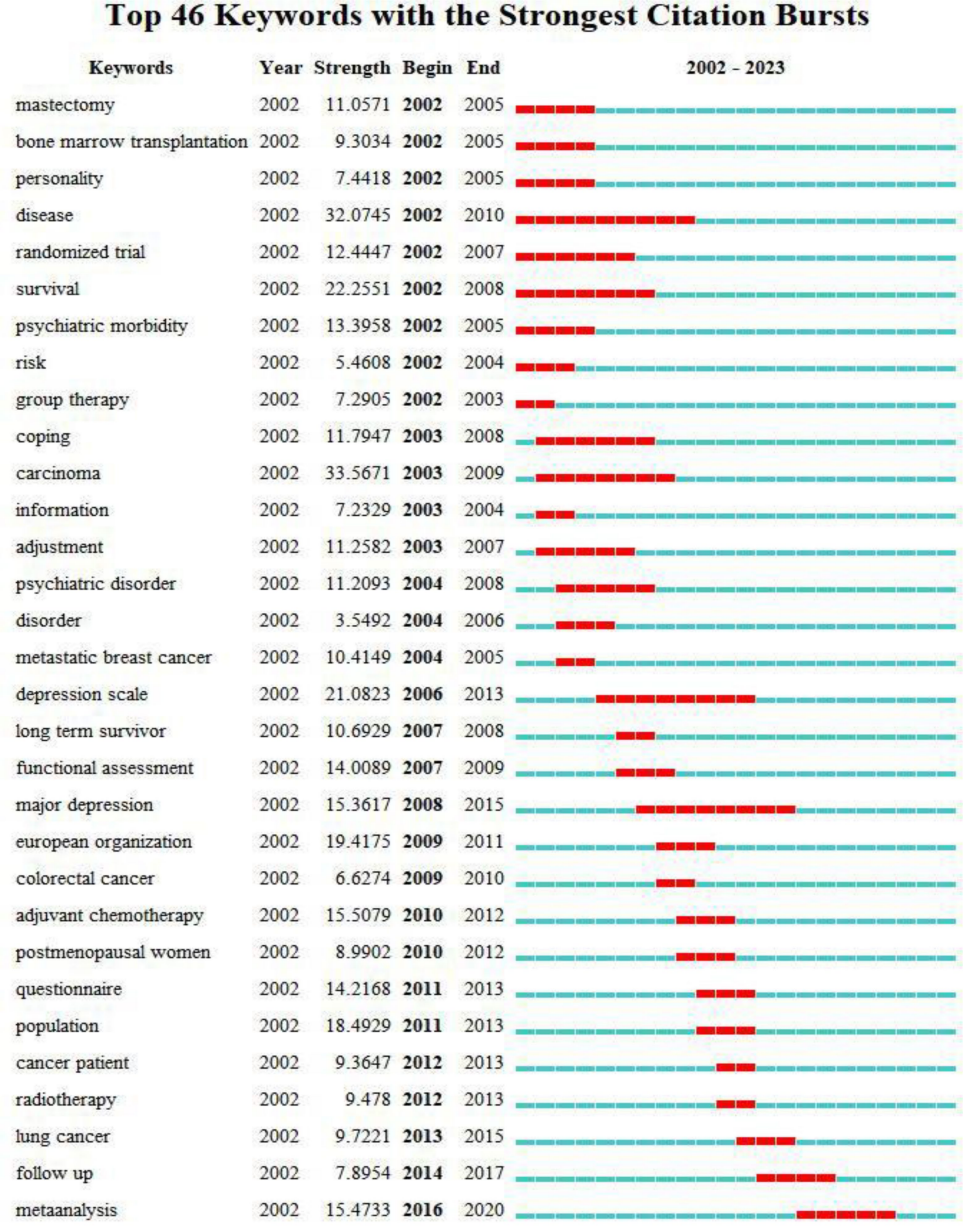
Keywords co-occurrence analysis of global research about depression in breast cancer. **(A)** Network visualization of the keywords; **(B)** Keywords co-occurrence overlay mapping from 2002 to 2022.

Keywords indicate research hotspots, core content, and developing trends In a field. The Top five most frequent keywords were breast cancer (1,570), depression (1,372), cancer (1,315), quality of life (971), and anxiety (754; Table 4). As shown in the network map (Figure 4A), the keywords can be divided into five clusters: supportive care and post-traumatic growth (in red) mindfulness and psychotherapy (in green) cognitive-behavioral therapy and cognitive impairment (in blue) physical activity and physical function (in yellow) symptom cluster and symptom distress (in purple). The development of keywords over time was explored within the depression in breast cancer (Figure 4B). The keywords in purple appeared earlier while the keywords in yellow appeared later.

**Table 4:** High-frequency keywords in the studies of depression in breast cancer.

Rank	Keywords	Frequency, n	Percentage, %	Cumulative percentage, %
1	Breast cancer	1,570	7.2	7.2
2	Depression	1,372	6.29	13.49
3	Cancer	1,315	6.03	19.52
4	Quality of life	971	4.45	23.97
5	Anxiety	754	3.46	27.43
6	Oncology	669	3.07	30.5
7	Fatigue	386	1.77	32.27
8	Chemotherapy	266	1.22	33.49
9	Distress	215	0.99	34.48
10	Psychological distress	212	0.97	35.45
11	Social support	199	0.91	36.36
12	Survivor ship	153	0.7	37.06
13	Breast neoplasms	147	0.67	37.73
14	Pain	140	0.64	38.37
15	Coping	139	0.64	39.01
16	Psycho-oncology	135	0.62	39.63
17	Exercise	130	0.6	40.23
18	Stress	127	0.58	40.81
19	Physical activity	123	0.56	41.37
20	Cancer survivors	119	0.55	41.92
21	Meta-analysis	116	0.53	42.45
22	Systematic review	108	0.5	42.95
23	Depressive symptoms	104	0.48	43.43
24	Sleep	102	0.47	43.9
25	Palliative care	98	0.45	44.35
26	Inflammation	94	0.43	44.78
27	Health-related quality of life	94	0.43	45.21
28	Symptoms	92	0.43	45.64
29	Mental health	92	0.43	46.07
30	Randomized controlled trial	80	0.37	46.44
31	Screening	80	0.37	46.81
32	Breast cancer survivors	80	0.37	47.18
33	Prostate cancer	76	0.35	47.53
34	Insomnia	74	0.34	47.87
35	Body image	73	0.33	48.2
36	Radiotherapy	73	0.33	48.53
37	Menopause	72	0.33	48.86
38	Neoplasms	71	0.33	49.19
39	Supportive care	70	0.32	49.51
40	Cancer-related fatigue	70	0.32	49.83
41	Survivors	69	0.32	50.15
42	Cancer survivorship	68	0.31	50.46
43	Mindfulness	67	0.31	50.77
44	Survival	67	0.31	51.08
45	Cytokines	66	0.3	51.38
46	Cognition	62	0.28	51.66
47	Lung cancer	60	0.28	51.94
48	Advanced cancer	60	0.28	52.22
49	Nursing	57	0.26	52.48
50	Mastectomy	57	0.26	52.74

### Burst of keywords

**Figure 5:**
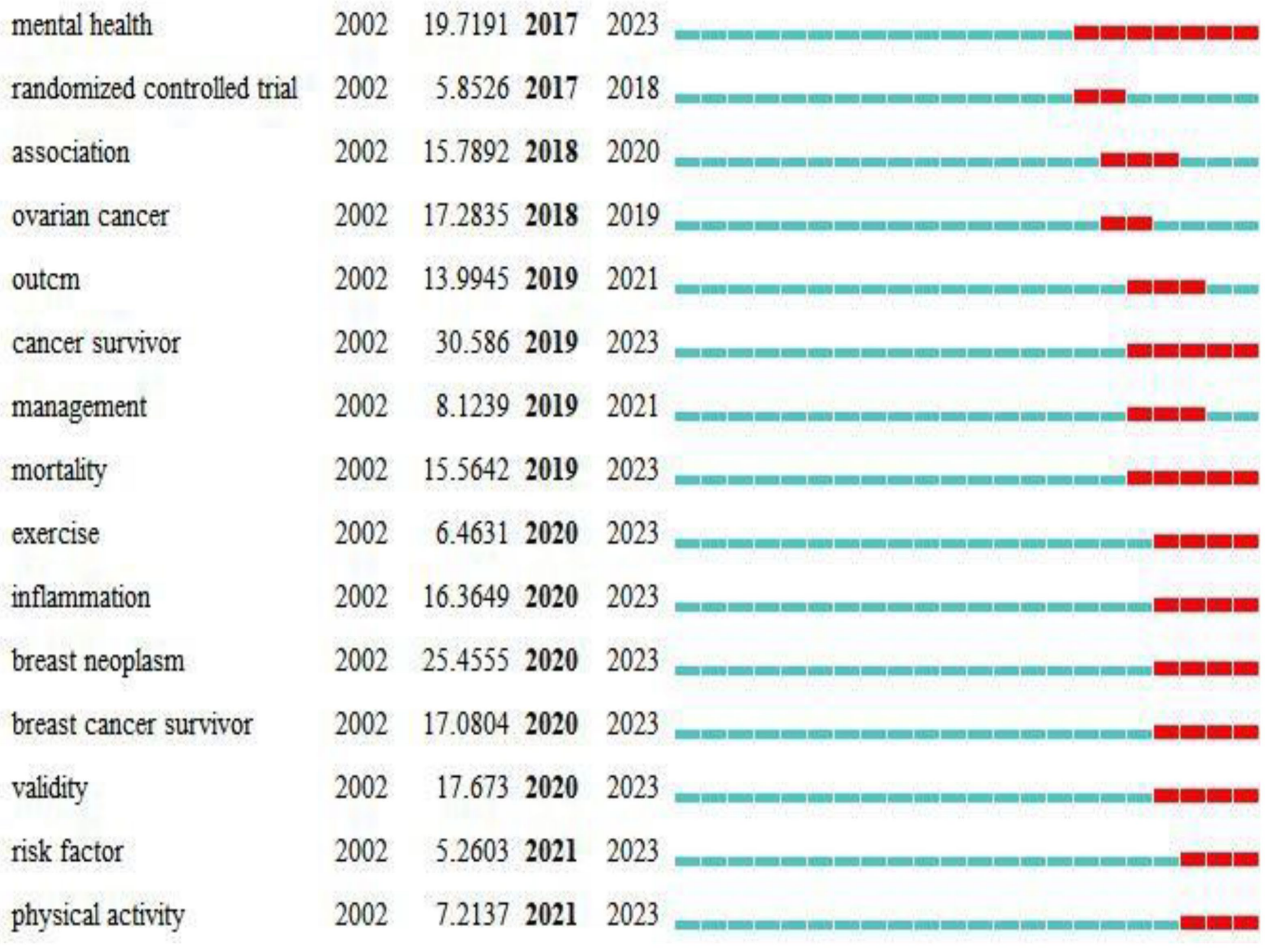
The evolution trend of depression in breast cancer from 2002 to 2020.

The burst of keywords reflects the sudden increase in keywords during a specific period, displays the time distribution and dynamic evolution of keywords, and accurately reveals the trends of research hotspots (Palesh et al., 2010; Qiu et al., 2013). The keywords with the strongest citation bursts were detected in the 7,350 publications using CiteSpace (Figure 5). The research frontiers included mental health (19.72), cancer survivor (30.59), mortality (15.56), exercise (6.46), and physical activity (7.21).

## Discussion

### Post-traumatic growth

The Chinese Psychosocial Oncology Therapy Guidelines for Cancer Patients, issued by the Chinese Psychosocial Oncology Society (CPOS) in 2016, pointed out that anxiety and depression are the major psychological problems in breast cancer patients, which may occur during the diagnosis, treatment, and rehabilitation periods. Anxiety and depression can affect the physical and mental development of cancer patients. Post-traumatic growth (PTG), positive psychological changes after encountering traumatic events, has become an important indicator to measure the psychological recovery of cancer patients. Anxiety and depression were proven to affect the PTG of breast cancer patients (Shi et al., 2021). Therefore, psychological interventions should be taken to reduce anxiety and depression, improve resilience, and promote PTG in breast cancer patients (Li et al., 2020). Studies suggested that the nurse-led supportive intervention relieved anxiety and depression, promoted post-traumatic growth, and facilitated recovery in breast cancer survivors (Xie et al., 2020; Wang et al., 2022).

### Psychological interventions

A series of mindfulness-based therapies have been gradually developed, such as mindfulness-based cognitive therapy (MBCT), acceptance and commitment therapy (ACT), and mindfulness-based stress reduction (MBSR). These therapies include mindful eating, body scan, mindful breathing, mindful meditation, and 3-min breathing space. Mindfulness-based stress reduction therapy is currently the most widely used psychological intervention in breast cancer worldwide. Breast cancer diagnosis and treatment may drastically affect the quality of life and cause depression and anxiety. Mindfulness-based interventions can effectively relieve stress, improve mental states including anxiety and depression, and improve the quality of life in breast cancer patients (Kenne Sarenmalm et al., 2017; Priya and Kalra, 2018). Jing et al. (2021) reported that mindfulness-based intervention improved overall outcomes among Chinese breast cancer patients. Recent research showed that a mindfulness-based stress reduction approach, group counseling, and survivorship education could reduce depressive symptoms in young breast cancer survivors (Mirmahmoodi et al., 2020; Bower et al., 2021; Printz, 2021). Mindfulness has also benefited survivors by eliminating cancer-related pain and cognitive impairment (Smith et al., 2021; Duval et al., 2022). It was proven that implementing the multi-modal mindfulness-based intervention, including integrative dietary, physical activity, and mindfulness, enhanced breast cancer survivors’ quality of life and healthy lifestyle (Ruiz-Vozmediano et al., 2020; Wang et al., 2022). In the future, mindfulness can be added to clinical health education for patients with breast cancer to promote their overall quality of life.

### Cognitive-behavioral therapy

Mastectomy can worsen body image and impact sexual performance among breast cancer patients. Therefore, their psychological problems are common, including increased heart rate, elevated blood pressure, tension, anxiety, fear, sleep disturbance, etc. These psychological problems can reduce patients’ immunity and affect surgery and postoperative rehabilitation. Computerized cognitive-behavioral therapy (CCBT) is an interactive computer interface with clear operation steps, web pages, comics, animations, videos, sounds, and other highly structured media interactions to modify how the patient thinks, believes, or behaves. The CCBT is designed to change the unhealthy cognitive structure and eliminate unhealthy emotions and behaviors. Studies confirmed that computerized cognitive-behavioral therapy could improve anxiety, depression, and sleep quality in preoperative breast cancer patients (Gorman et al., 2021).

### Physical activity

Physical activity benefits breast cancer patients during treatment and survivorship. Evidence suggests that regular physical activity after breast cancer diagnosis reduces early all-cause mortality, breast cancer mortality, and cancer recurrence. In addition, regular moderate-to-vigorous physical activity can reduce cancer-related fatigue and depression and improve physical function during adjuvant treatment. One study showed that a 10-week physical exercise intervention significantly improved psychosocial well-being (Mehnert et al., 2011). Furthermore, postoperative standardized and effective functional exercise can reduce the occurrence of postoperative edema. Despite substantial evidence supporting physical activity’s benefits during cancer survivorship, most breast cancer patients do not adhere to the national exercise guidelines. Women with depressive symptoms feel exercise is challenging, but these self-perceptions do not reflect their physical condition. After breast cancer surgery, depression screening tools can be used to identify individuals with decreased physical activity during survivorship and to determine when to intervene.

### Symptom cluster management

Breast cancer surgery and chemoradiation therapy can cause complications such as fatigue, pain, sleep disturbance, anxiety, and depression. Fatigue and pain are closely related, and pain can cause sleep disorders. Furthermore, sleep disorders are the main predisposing factors for anxiety, depression, and fatigue. These symptoms are interrelated, resulting in a synergistic effect of the symptom clusters, exacerbating the difficulty of patients’ recovery. Clinical studies have shown that rehabilitation exercises combined with acupoint massage can stimulate neuron regeneration and neurotrophic factor secretion, increase muscle strength and activity, improve various accompanying symptoms and improve quality of life. However, acupoint massage requires manual operation, making it difficult for patients to implement. On the other hand, there are significant individual differences in empirical treatment programs, and the long-term effects are challenging to evaluate and verify. The AR somatosensory acupoint massage is more effective in improving patients’ symptoms. Gradually increasing intervention time can further improve patients’ learning experience, encouraging them to actively complete training tasks and significantly improve their compliance with self-rehabilitation. Breast cancer patients receiving aromatase inhibitor therapy suffer various symptoms, which are affected by many factors such as anxiety, depression, educational level, and history of chemotherapy. Medical staff should pay attention to the patient’s psychological state, educational level, and history of chemotherapy during treatment and make decisions accordingly. Scientific, targeted, and individualized intervention programs should be developed to improve the patient’s symptoms.

## Strengths and limitations

According to our knowledge, this study is the first bibliometric analysis of depression in breast cancer. It can provide a historical perspective for future research and highlight research areas requiring further investigation. In addition, before the literature retrieval, we read many high-level papers and extracted search terms related to depression in breast cancer. After integration, we formulated the search strategy for this study. Therefore, the search strategy is complete and scientific. Meanwhile, this study has some shortcomings. The WOS database, which owns standardized and comprehensive records for bibliometric analysis, is considered the most reliable data source. Therefore, we only searched this. Some studies outside this database may have been overlooked. In addition, this study has many authors, some of whom may have changed names or researched from multiple institutions. Although we have carefully checked the process, some mistakes are inevitable.

## Data availability statement

The original contributions presented in the study are included in the article/supplementary material, further inquiries can be directed to the corresponding author.

## Author contributions

LC wrote the manuscript. TR designed and guided the research. YT analyzed the data. HL prepared the figures and tables. All authors contributed to the article and approved the submitted version.

## Funding

This study was funded by the Guizhou Provincial Health and Health Commission (Gzwkj2022-244), the Nursing Hospital Fund Project of Affiliated Hospital of Guizhou Medical University (Project No. GYHLB202203), and the Nursing Hospital Fund Project of Affiliated Hospital of Guizhou Medical University (Grant No. GYHLB202222).

## Conflict of interest

The authors declare that the research was conducted in the absence of any commercial or financial relationships that could be construed as a potential conflict of interest.

## Publisher’s note

All claims expressed in this article are solely those of the authors and do not necessarily represent those of their affiliated organizations, or those of the publisher, the editors and the reviewers. Any product that may be evaluated in this article, or claim that may be made by its manufacturer, is not guaranteed or endorsed by the publisher.
